# Association of heart rate variability with preoperative acute insomnia in patients scheduled for elective surgery

**DOI:** 10.3389/fneur.2025.1513395

**Published:** 2025-05-07

**Authors:** Zhenqiao Zhao, Junchao Liang, Shujie Hou, Guojia Zhu, Ning Liu, Wei Hao, Zhijuan Xu

**Affiliations:** ^1^Graduate School of Hebei University of Traditional Chinese Medicine, Shijiazhuang, Hebei, China; ^2^Department of Anesthesiology, Hebei Provincial Hospital of Traditional Chinese Medicine, Hebei Technology Innovation Center of TCM Spleen and Kidney Diseases, Shijiazhuang, Hebei, China; ^3^Office of Physician-Patient Communication, Hebei Provincial Hospital of Traditional Chinese Medicine, Shijiazhuang, Hebei, China; ^4^Department of Hepatobiliary Surgery, The Second Hospital of Hebei Medical University, Shijiazhuang, Hebei, China

**Keywords:** preoperative acute insomnia, heart rate variability, predictive model, receiver operating characteristic, nomogram

## Abstract

**Objective:**

Heart rate variability (HRV), which reflects the balance of the sympathetic and parasympathetic systems, is associated with insomnia. However, its relationship with preoperative acute insomnia has not yet been investigated. This study aimed to assess the associations of HRV characteristics with preoperative acute insomnia.

**Methods:**

This study enrolled 563 patients who were scheduled for elective surgery. Preoperative clinical characteristics, including demographics, the apnea–hypopnea index (AHI), HRV characteristics, and sleep quality data, were recorded.

**Results:**

Among the 563 patients included, 78.5% met the criteria for insomnia. Age (*P* = 0.005), AHI score (*P* < 0.001), and AHI stage (*P* < 0.001) were positively associated, whereas education level (*P* = 0.004) was negatively associated with preoperative acute insomnia. In terms of HRV characteristics, low-frequency (LF) (*P* = 0.012) and high-frequency (HF) (*P* = 0.011) were positively associated with preoperative acute insomnia. Multivariate logistic regression analyses screened out the variables associated with preoperative acute insomnia, including education level [*P* = 0.028, odds ratio (OR) = 0.603], AHI score (*P* < 0.001, OR = 1.068), standard deviation of all normal NN intervals (SDNN) (*P* = 0.004, OR = 0.956), the root mean square of the successive differences (rMSSD) (*P*= 0.001, OR = 1.130), NN50 count divided by the total number of all NN intervals (pNN50) (*P* = 0.006, OR = 0.893), ultra-low-frequency (ULF) (*P* = 0.003, OR = 1.000), LF/HF ratio (*P* = 0.018, OR = 0.608), and HF ratio (*P* = 0.072, OR = 0.953). Receiver operating characteristic analysis revealed that the combination of these variables had good predictive value for preoperative acute insomnia, with an area under the curve of 0.750.

**Conclusion:**

Preoperative acute insomnia is a prevalent issue and is associated with an imbalance in the sympathetic/parasympathetic system. A predictive model based on HRV characteristics may improve the management of preoperative acute insomnia.

## 1 Introduction

Acute insomnia is characterized by prolonged time to fall asleep, low sleep quality, and reduced duration of sleep, with symptoms lasting less than a month (1). In clinical practice, preoperative acute insomnia, the acute onset of insomnia on the night before surgery, is commonly observed (2–4). In recent years, the interest in preoperative acute insomnia has increased. Previous studies have reported that preoperative acute insomnia exacerbates surgery-induced neuroinflammation and neuronal damage and is associated with postoperative pain and postoperative delirium, which strongly affects the recovery and quality of life of patients (5–8). Therefore, the management of preoperative acute insomnia should be improved, and seeking predictors for preoperative acute insomnia may be a potential solution.

Heart rate variability (HRV), which involves the time domain and frequency domain, reflects the activation of the sympathetic and parasympathetic systems, as well as their balance (9). Previous studies have reported that HRV characteristics are associated with insomnia (10–13). For example, it has been reported that high-frequency (HF) sleep is lower in patients with insomnia than in normal sleepers (10). Another study revealed that lower HF during worry induction was able to predict insomnia prospectively (11). Several interventional studies used HRV biofeedback to regulate autonomic balance and reported that this intervention improved insomnia (12, 13). Therefore, it could be assumed that HRV characteristics are closely associated with preoperative acute insomnia. However, there is no evidence.

Cardiopulmonary coupling (CPC) is a wearable device used to monitor HRV and sleep quality. This device is convenient and portable and has been widely used for investigating sleep problems (14, 15). Our previous study used CPC to explore the prevalence and risk factors for preoperative obstructive sleep apnea in patients with plans to receive surgery under general anesthesia (16). The present study aimed to assess the associations of HRV characteristics with preoperative acute insomnia using CPC and construct a predictive model for preoperative acute insomnia on the basis of demographics and HRV characteristics.

## 2 Methods

### 2.1 Patients

A total of 563 patients scheduled for elective major surgery at Hebei Provincial Hospital of Traditional Chinese Medicine between October 2022 and December 2023 were consecutively enrolled in this study. The inclusion criteria were as follows: (1) scheduled for elective major surgery; (2) aged more than 18 years; (3) cooperated with wearing CPC for sleep quality and HRV evaluation; and (4) without a history of chronic insomnia. The exclusion criteria were as follows: (1) complicated with cardiac arrhythmias; (2) had implantation of a heart pacemaker; (3) failed to acquire data via the CPC assessment system; and (4) pregnant females. Approval for the study was obtained from the Ethics Committee (No. 2020-KY-067-02). All patients provided signed informed consent.

### 2.2 Data collection

Each patient wore the CPC device for a single night before the operation. Preoperative clinical characteristics, including demographics, apnea–hypopnea index (AHI) scores, and preoperative anxiety status, were recorded. The perioperative anxiety scale-7 (PAS-7) and visual analog scale for anxiety (VAS-A) were used to evaluate the preoperative anxiety status of patients (17). In addition, preoperative sleep quality data were also gathered. Based on the sleep quality data, patients were defined as having preoperative acute insomnia if they met one of the following criteria: 1. Prolonged sleep latency: the time to fall asleep exceeds 30 min; 2. The symptoms of sleep maintenance disorders: more than two awakenings at night or early morning awakening; 3. Decreased sleep quality: shallow sleep and frequent dreams; 4. Shortened total sleep time: usually < 6 h; 5. Daytime residual effects: feeling dizzy, listless, sleepy, and weak the next morning.

HRV features, which included the standard deviation of all the normal NN intervals (SDNN), standard deviation of 5 min average normal NN intervals (SDANN), the root mean square of the successive differences (rMSSD), heart rate variability triangular index (HRVTI), NN10 count divided by the total number of all NN intervals (pNN10), NN20 count divided by the total number of all NN intervals (pNN20), NN30 count divided by the total number of all NN intervals (pNN30), NN40 count divided by the total number of all NN intervals (pNN40), NN50 count divided by the total number of all NN intervals (pNN50), ultra-low-frequency (ULF), very-low-frequency (VLF), low-frequency (LF), HF, LF/HF ratio, LF ratio, and HF ratio, were collected.

### 2.3 Model and evaluation

Multivariate logistic regression analyses were used to identify factors related to preoperative acute insomnia risk. Considering the high correlation between variables only one representative variable was included (such as among HF, LF, and LF/HF ratio, the latter was retained in the model; between AHI score and AHI stage, the former retained; between SDNN and SDANN, the former retained; among pNN10 pNN20, pNN30 pNN40, and pNN50, pNN50 was retained). After the redundant variables were eliminated, the variables included in the multivariate logistic regression analyses were age, gender, BMI, education level, hypertension, diabetes, surgical sites, AHI score, PAS-7, VAS-A, SDNN, rMSSD, HRVTI, pNN50, ULF, VLF, LF/HF ratio, LF ratio, and HF ratio. Considering that there might still be some indirect correlation between the retained variables, backward stepwise regression was used. The purpose was to allow the model to automatically select the variables with significant contributions through an iterative approach to minimize the influence of multicollinearity on the generalizability of the model. Receiver operating characteristic (ROC) analyses were used to evaluate the diagnostic utility of factors for preoperative acute insomnia. The Hosmer–Lemeshow test was used to confirm the model performance.

### 2.4 Nomogram construction

RStudio software with R version 4.3.3 (https://www.r-project.org/) was used to construct the nomograms. The “rms” package was used to construct combined models, and the “regplot” package was used to construct nomograms. Nomograms provided a visual representation of the combined models, in which all the factors selected by multivariate logistic regression analyses were included. The regression coefficients were scaled and translated into points on the nomogram, which allowed for a user-friendly graphical interface to estimate preoperative acute insomnia risk. The probability of preoperative acute insomnia risk was calculated as odds/(odds+1) (18).

### 2.5 Statistical analyses

SPSS version 26.0 (IBM, USA) was used for the statistical analyses. This study did not conduct a sample size calculation but rather enrolled as many patients as possible. Descriptive analyses were conducted to summarize the clinical characteristics and HRV features. Continuous variables are described as the means ± standard deviations (SDs), and categorical variables are described as frequencies with percentages. Comparisons between patients with and without preoperative acute insomnia were performed via Student's *t* test, *the chi*-square test, and the Wilcoxon rank sum test, as appropriate. The detailed expansions were as follows: (1) continuous variables of clinical characteristics and HRV features were compared via Student's t test; (2) unordered categorical variables of clinical characteristics, such as sex (female vs. male), were compared via *the chi*-square test; and (3) ordered categorical variables, such as the AHI stage (none < mild < moderate < severe), were compared via the Wilcoxon rank sum test. All the statistical analyses used a two-tailed test, with a *P* value < 0.05 indicating significance.

## 3 Results

### 3.1 Study flow

**Figure 1:**
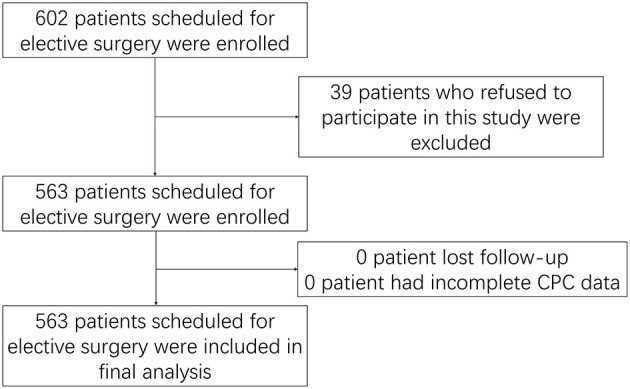
Study flow.

A total of 602 patients scheduled for elective surgery were enrolled, and 39 of them were excluded because they refused to participate in this study. A total of 563 patients were subsequently included in this study. Since the patients wore the CPC device for a single night before the operation, no lost-to-follow-up or incomplete CPC data occurred. Finally, all 563 patients were included in the analysis (Figure 1).

### 3.2 Clinical and HRV characteristics

There were 319 (56.7%) females and 224 (43.3%) males included in this study, with a mean age of 54.2 ± 15.1 years. The surgical sites were the head and neck in 111 (19.7%) patients, the chest in 102 (18.1%) patients, the abdomen in 124 (22.0%) patients, and the pelvic cavity in 226 (40.1%) patients. The mean AHI score was 14.8 ± 14.7. Moreover, the mean PAS-7 and VAS-A scores were 9.9 ± 2.5 and 4.5 ± 1.4, respectively. The other information is listed in Table 1.

**Table 1:** Clinical characteristics of patients.

**Characteristics**	**Patients (N = 563)**
Age (years), mean ± SD	54.2 ± 15.1
**Sex**, ***n*** **(%)**	
Female	319 (56.7)
Male	244 (43.3)
BMI (kg/m^2^), mean ± SD	24.8 ± 3.8
**Education level**, ***n*** **(%)**	
Below high school	351 (62.3)
High school or above	212 (37.7)
**Hypertension**, ***n*** **(%)**	
No	419 (74.4)
Yes	144 (25.6)
**Diabetes**, ***n*** **(%)**	
No	492 (87.4)
Yes	71 (12.6)
**Surgical sites**, ***n*** **(%)**	
Head and neck	111 (19.7)
Chest	102 (18.1)
Abdomen	124 (22.0)
Pelvic cavity	226 (40.1)
AHI score, mean ± SD	14.8 ± 14.7
**AHI stage**, ***n*** **(%)**	
None (AHI score < 5)	160 (28.4)
Mild (5 ≤ AHI score < 15)	189 (33.6)
Moderate (15 ≤ AHI score < 30)	135 (24.0)
Severe (AHI score ≥30)	79 (14.0)
PAS-7 score, mean ± SD	9.9 ± 2.5
VAS-A score, mean ± SD	4.5 ± 1.4

SD, standard deviation; BMI, body mass index; AHI, apnea-hypopnea index; PAS-7, perioperative anxiety scale-7; VAS-A, visual analog scale for anxiety.

The HRV characteristics are listed in detail in Table 2. With respect to the time domain, the mean SDNN, SDANN, and rMSSD values were 116.0 ± 34.9 ms, 98.5 ± 32.0 ms, and 31.2 ± 17.0 ms, respectively. In terms of the frequency domain, the mean ULF, LF/HF ratio, and HF ratio were 14520.8 ± 10497.0 ms^2^, 1.6 ± 0.8, and 16.6 ± 8.5, respectively.

**Table 2:** HRV features.

**Features**	**Patients (*N* = 563)**
**Time domain**	
SDNN (ms), mean ± SD	116.0 ± 34.9
SDANN (ms), mean ± SD	98.5 ± 32.0
rMSSD (ms), mean ± SD	31.2 ± 17.0
HRVTI (ms), mean ± SD	17.9 ± 7.1
pNN10 (%), mean ± SD	57.4 ± 15.8
pNN20 (%), mean ± SD	38.2 ± 17.8
pNN30 (%), mean ± SD	26.0 ± 16.7
pNN40 (%), mean ± SD	13.7 ± 13.2
pNN50 (%), mean ± SD	10.0 ± 11.4
**Frequency domain**	
ULF (ms^2^), mean ± SD	14520.8 ± 10497.0
VLF (ms^2^), mean ± SD	2576.7 ± 11707.6
LF (ms^2^), mean ± SD	808.6 ± 1080.5
HF (ms^2^), mean ± SD	654.4 ± 885.1
LF/HF ratio, mean ± SD	1.6 ± 0.8
LF ratio, mean ± SD	21.0 ± 5.3
HF ratio, mean ± SD	16.6 ± 8.5

HRV, heart rate variability; SD, standard deviation; SDNN, SD of all normal NN intervals; SDANN, SD of 5 minutes average normal NN intervals; rMSSD, the root mean square of the successive differences; HRVTI, heart rate variability triangular index; pNN10, NN10 count divided by the total number of all NN intervals; pNN20, NN20 count divided by the total number of all NN intervals; pNN30, NN30 count divided by the total number of all NN intervals; pNN40, NN40 count divided by the total number of all NN intervals; pNN50, NN50 count divided by the total number of all NN intervals; ULF, ultra-low-frequency; VLF, very-low-frequency; LF, low-frequency; HF, high-frequency.

### 3.3 Incidence of preoperative acute insomnia

**Figure 2:**
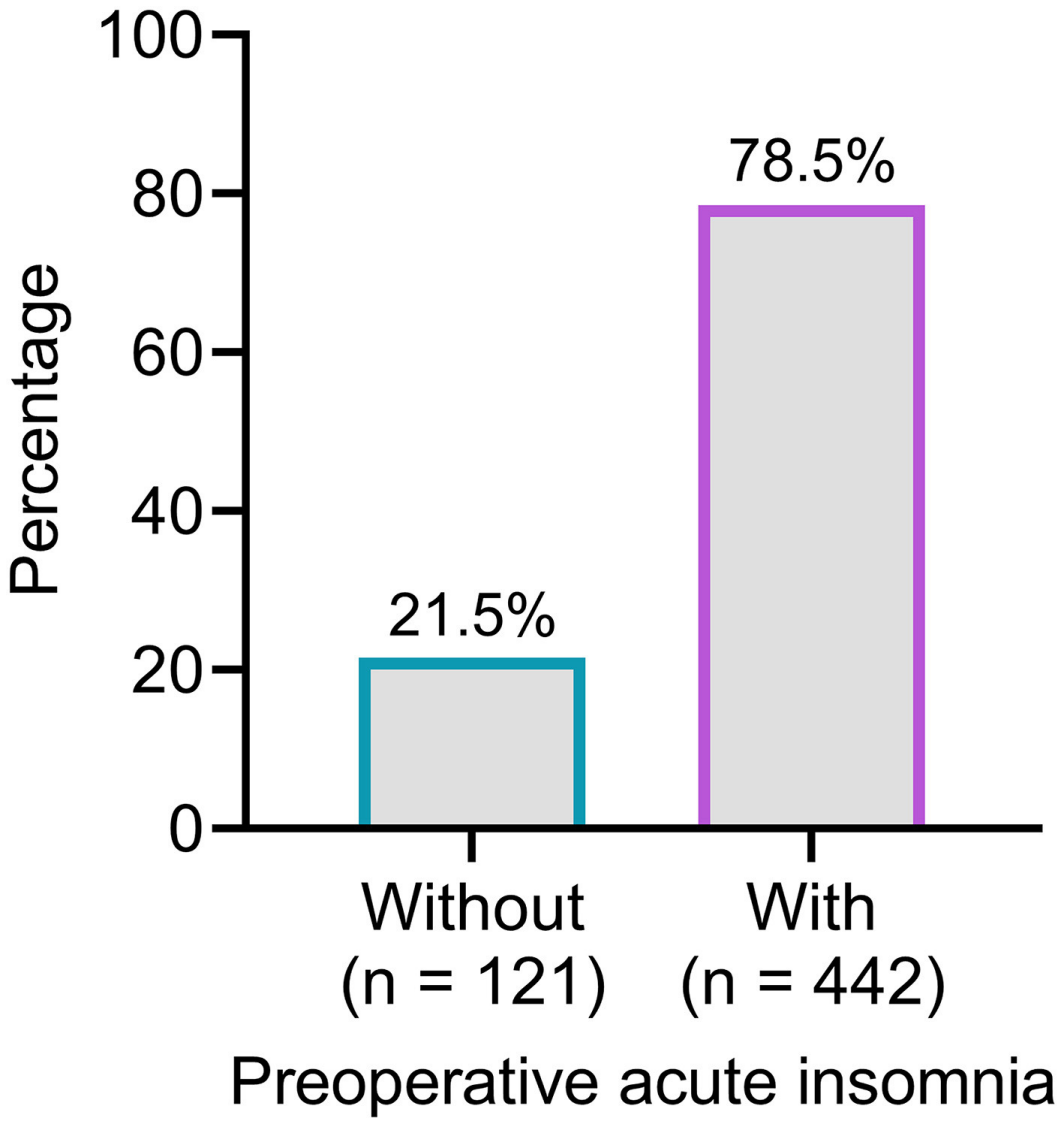
The number and proportion of patients with and without preoperative acute insomnia. A total of 442 (78.5%) patients were identified with preoperative acute insomnia.

Preoperative acute insomnia was identified in 442 patients, whereas the other 121 patients did not have preoperative acute insomnia. Therefore, the incidence of preoperative acute insomnia was 78.5% (Figure 2).

### 3.4 Association of clinical and HRV characteristics with preoperative acute insomnia

Age (*P* = 0.005), AHI score (*P* < 0.001), and AHI stage (*P* < 0.001) were positively associated with preoperative acute insomnia, whereas education level (*P* = 0.004) was negatively associated with preoperative acute insomnia. However, sex, BMI, hypertension, diabetes, surgical site, PAS-7 score, and VAS-A score were not associated with preoperative acute insomnia (all *P* > 0.05).

In terms of HRV characteristics, LF (*P* = 0.012) and HF (*P* = 0.011) were positively associated with preoperative acute insomnia. The other HRV characteristics were not associated with preoperative acute insomnia (all *P* > 0.05) (Table 3).

**Table 3:** Correlation of clinical characteristics and HRV features with preoperative acute insomnia risk.

**Items**	**Preoperative acute insomnia**		***P* value**
	**No (*****n*** = **121)**	**Yes (*****n*** = **442)**	
**Clinical characteristics**			
Age (years), mean ± SD	50.8 ± 14.8	55.2 ± 15.1	0.005
**Sex**, ***n*** **(%)**			0.613
Female	71 (58.7)	248 (56.1)	
Male	50 (41.3)	194 (43.9)	
BMI (kg/m^2^), mean ± SD	24.9 ± 3.4	24.8 ± 4.0	0.689
**Education level**, ***n*** **(%)**			0.004
Below high school	62 (51.2)	289 (65.4)	
High school or above	59 (48.8)	153 (34.6)	
**Hypertension**, ***n*** **(%)**			0.647
No	92 (76.0)	327 (74.0)	
Yes	29 (24.0)	115 (26.0)	
**Diabetes**, ***n*** **(%)**			0.485
No	108 (89.3)	384 (86.9)	
Yes	13 (10.7)	58 (13.1)	
**Surgical sites**, ***n*** **(%)**			0.800
Head and neck	25 (20.7)	86 (19.5)	
Chest	19 (15.7)	83 (18.8)	
Abdomen	25 (20.7)	99 (22.4)	
Pelvic cavity	52 (43.0)	174 (39.4)	
AHI score, mean ± SD	8.7 ± 11.3	16.4 ± 15.1	< 0.001
**AHI stage**, ***n*** **(%)**			< 0.001
None (AHI score < 5)	57 (47.1)	103 (23.3)	
Mild (5 ≤ AHI score < 15)	41 (33.9)	148 (33.5)	
Moderate (15 ≤ AHI score < 30)	15 (12.4)	120 (27.1)	
Severe (AHI score ≥30)	8 (6.6)	71 (16.1)	
PAS-7 score, mean ± SD	10.2 ± 2.6	9.8 ± 2.5	0.165
VAS-A score, mean ± SD	4.6 ± 1.2	4.5 ± 1.4	0.204
**HRV features**			
SDNN (ms), mean ± SD	114.4 ± 30.0	116.5 ± 36.2	0.561
SDANN (ms), mean ± SD	97.0 ± 27.2	98.9 ± 33.2	0.570
rMSSD (ms), mean ± SD	30.0 ± 12.5	31.5 ± 18.1	0.372
HRVTI (ms), mean ± SD	18.0 ± 6.4	17.9 ± 7.3	0.822
pNN10 (%), mean ± SD	59.0 ± 15.3	57.0 ± 15.9	0.214
pNN20 (%), mean ± SD	39.2 ± 17.5	37.9 ± 17.8	0.464
pNN30 (%), mean ± SD	26.6 ± 16.5	25.8 ± 16.8	0.659
pNN40 (%), mean ± SD	14.1 ± 12.2	13.6 ± 13.4	0.712
pNN50 (%), mean ± bghzxSD	10.1 ± 10.0	10.0 ± 11.7	0.926
ULF (ms^2^), mean ± SD	13252.6 ± 7470.5	14868.0 ± 11165.7	0.062
VLF (ms^2^), mean ± SD	4200.2 ± 25127.6	2132.3 ± 1395.6	0.367
LF (ms^2^), mean ± SD	667.6 ± 475.0	847.2 ± 1191.4	0.012
HF (ms^2^), mean ± SD	530.2 ± 460.4	688.3 ± 967.1	0.011
LF/HF ratio, mean ± SD	1.6 ± 1.1	1.5 ± 0.8	0.426
LF ratio, mean ± SD	20.7 ± 4.7	21.0 ± 5.5	0.489
HF ratio, mean ± SD	16.4 ± 8.2	16.6 ± 8.5	0.838

HRV, heart rate variability; SD, standard deviation; BMI, body mass index; AHI, apnea-hypopnea index; PAS-7, perioperative anxiety scale-7; VAS-A, visual analog scale for anxiety; SDNN, SD of all normal NN intervals; SDANN, SD of 5 minutes average normal NN intervals; rMSSD, the root mean square of the successive differences; HRVTI, heart rate variability triangular index; pNN10, NN10 count divided by the total number of all NN intervals; pNN20, NN20 count divided by the total number of all NN intervals; pNN30, NN30 count divided by the total number of all NN intervals; pNN40, NN40 count divided by the total number of all NN intervals; pNN50, NN50 count divided by the total number of all NN intervals; ULF, ultra-low-frequency; VLF, very-low-frequency; LF, low-frequency; HF, high-frequency.

### 3.5 Construction of a model to predict preoperative acute insomnia

Multivariate logistic regression analyses screened out the variables associated with preoperative acute insomnia, including education level [*P* = 0.028, odds ratio (OR) = 0.603), AHI score (*P* < 0.001, OR = 1.068], SDNN (*P* = 0.004, OR = 0.956), rMSSD (*P* = 0.001, OR = 1.130), pNN50 (*P* = 0.006, OR = 0.893), ULF (*P* = 0.003, OR = 1.000), LF/HF ratio (*P* = 0.018, OR = 0.608), and HF ratio (*P* = 0.072, OR = 0.953) (Table 4).

**Table 4:** Multivariate logistic regression analyses for preoperative acute insomnia risk.

**Factors**	***P* value**	**OR**	**95% CI**	
			**Lower**	**Upper**
Education level	0.028	0.603	0.384	0.947
AHI score	< 0.001	1.068	1.040	1.096
SDNN	0.004	0.956	0.928	0.986
rMSSD	0.001	1.130	1.052	1.213
pNN50	0.006	0.893	0.824	0.968
ULF	0.003	1.000	1.000	1.000
LF/HF ratio	0.018	0.608	0.403	0.917
HF ratio	0.072	0.953	0.904	1.004

OR, odds ratio; CI, confidence interval; AHI, apnea-hypopnea index; SDNN, SD of all normal NN intervals; rMSSD, the root mean square of the successive differences; pNN50, NN50 count divided by the total number of all NN intervals; ULF, ultra-low-frequency; LF, low-frequency; HF, high-frequency.

**Figure 3:**
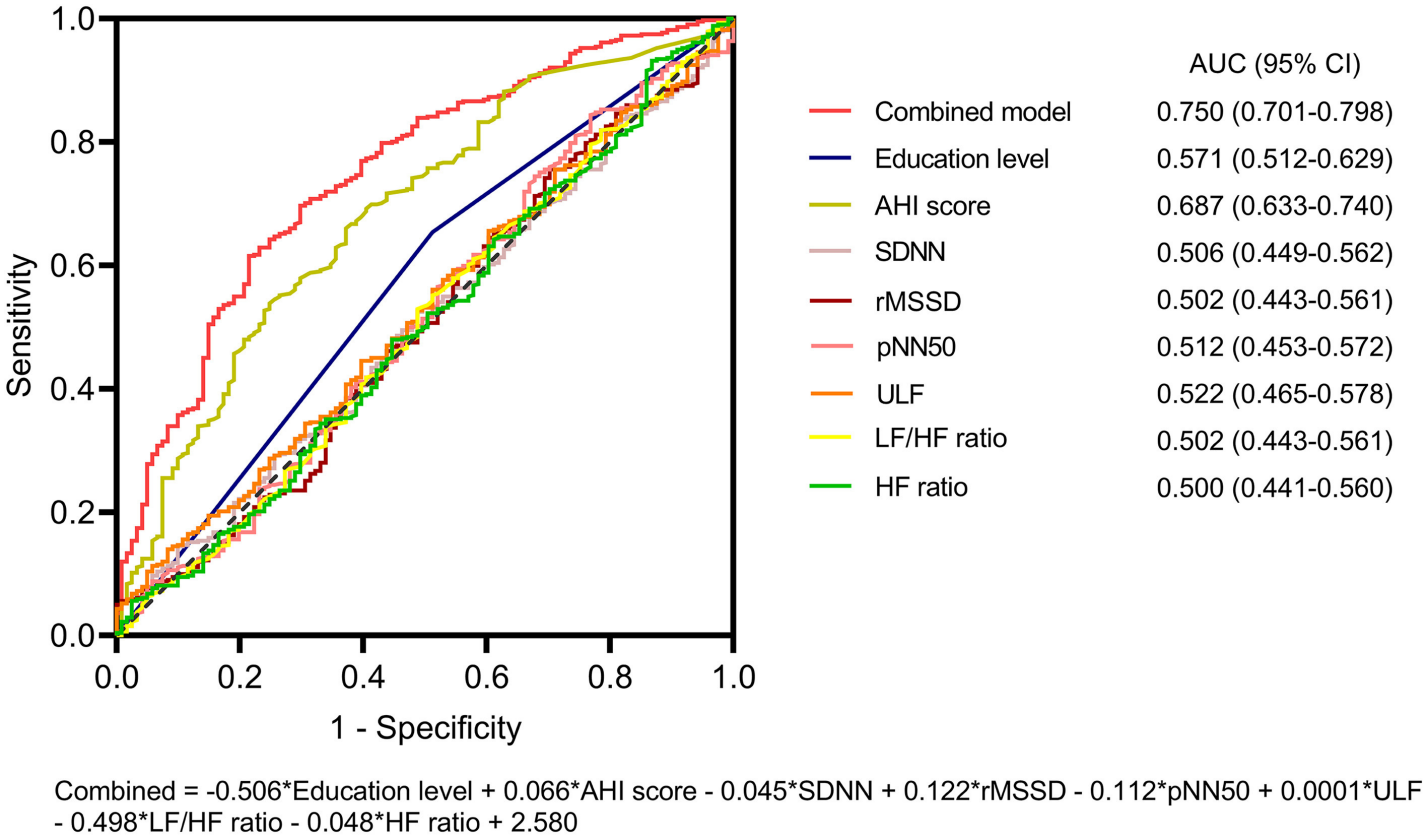
ROC analysis of the predictive model for preoperative acute insomnia. ROC curves of education level, the AHI score, the SDNN, the rMSSD, the pNN50, the ULF, the LF/HF ratio, the HF ratio, and the combined model for predicting preoperative acute insomnia. The combined model had the highest AUC for predicting preoperative acute insomnia.

Then, each of the variables and their combination for the prediction of preoperative acute insomnia were assessed via ROC analyses. The AHI score had an acceptable value for predicting preoperative acute insomnia [area under the curve (AUC) = 0.687, 95% confidence interval (CI) = 0.633–0.740], whereas the other variables had poor values for predicting preoperative acute insomnia (all AUCs < 0.6). However, the combination of these variables had good value for predicting preoperative acute insomnia (AUC = 0.750, 95% CI = 0.701–0.798) (Figure 3). According to the Hosmer–Lemeshow test, the model showed a good performance (*P* = 0.218).

### 3.6 Nomogram for predicting preoperative acute insomnia

**Figure 4:**
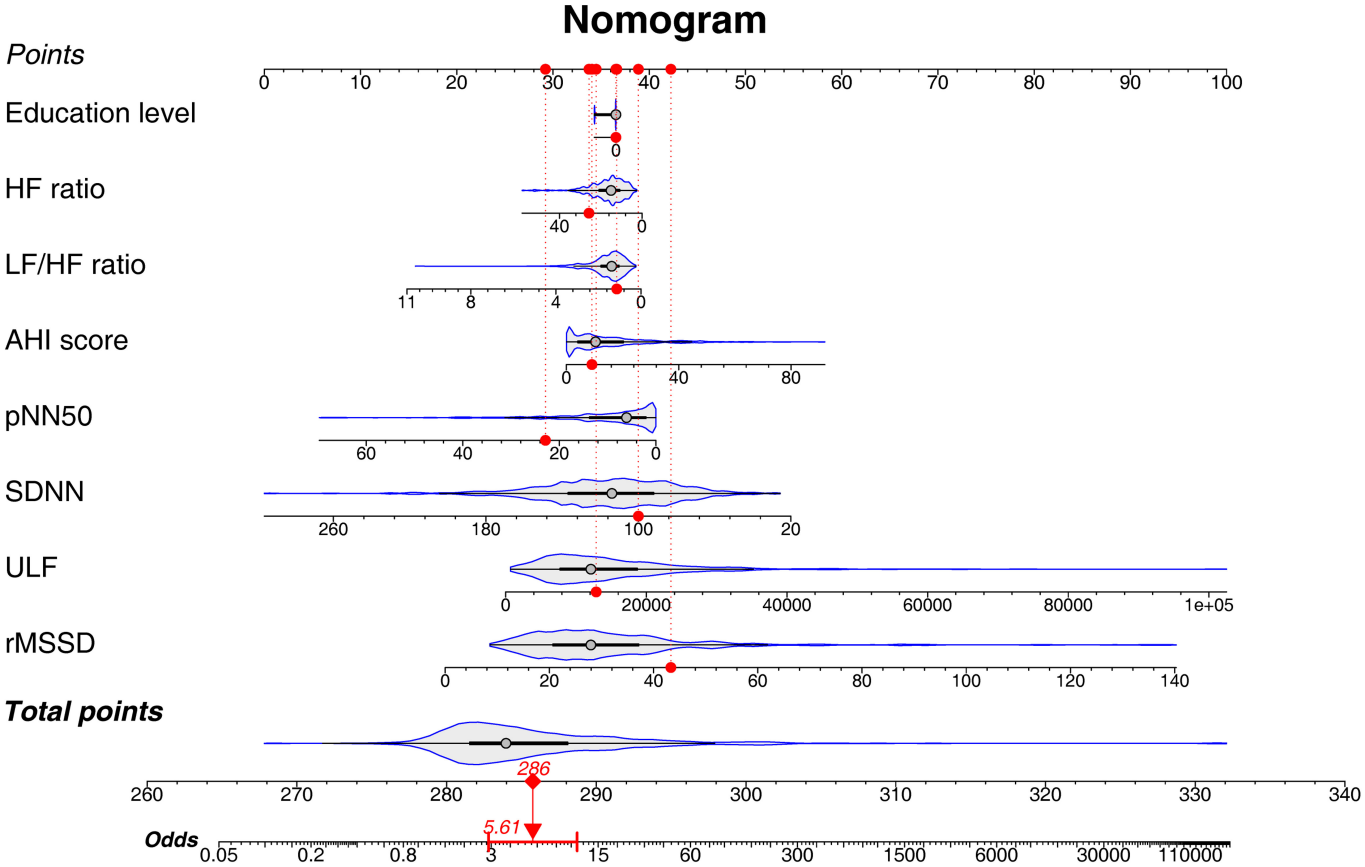
Nomogram of the predictive model for preoperative acute insomnia. A nomogram was constructed on the basis of education level, HF ratio, LF/HF ratio, AHI score, pNN50, SDNN, ULF, and rMSSD. This nomogram could be used by clinicians in practice with the following step-by-step instructions: 1. Gathering the information of a patient, including education level, HF ratio, LF/HF ratio, AHI score, pNN50, SDNN, ULF, and rMSSD. 2. Filling in each characteristics scale bar with the gathered information and marking a dot on the scale bar. 3. Projecting each dot to the Points scale bar at the top of the graph to get the point of each characteristic. 4. Calculating the sum of total points and marking a dot on the Total points scale bar at the bottom of the graph. 5. Projecting the total point dot to the Odds scale bar to get the odds of preoperative acute insomnia of the patient. 6. Calculating the risk of preoperative acute insomnia: The probability of preoperative acute insomnia risk = odds/(odds + 1).

A nomogram based on the combination of education level, HF ratio, LF/HF ratio, AHI score, pNN50, SNDD, ULF, and the rMSSD for predicting preoperative acute insomnia was constructed. The details of the nomogram are shown in Figure 4. A patient is given as an example, and the characteristics are marked as red dots in the nomogram. This patient had an education level of below high school, AHI score of 9.1, SDNN of 100.04 ms, rMSSD of 43.33 ms, pNN50 of 22.88%, ULF of 12865.1 ms^2^, LF/HF ratio of 1.14, and HF ratio of 25.66. According to the nomogram, the total points were 286 and the odds value was 5.61. The calculated risk of preoperative acute insomnia was 84.9%.

## 4 Discussion

The prevalence of preoperative acute insomnia varies greatly across studies (4, 6, 19–21). For example, Wu et al. (6) reported that in patients who had rotator cuff injury and underwent shoulder arthroscopy, the prevalence of low sleep quality, defined as a Pittsburgh Sleep Quality Index (PSQI) >7, was 70.1% (61/87). Bjurström et al. (4) used a cutoff value of PSQI > 5 and reported that the prevalence of preoperative sleep disturbance in patients with disabling osteoarthritis who underwent total hip arthroplasty was 73.1% (38/52). Ida et al. (19) reported that acute sleep disturbance prior to surgery, which was defined as < 85% preoperative sleep efficiency, occurred in 79.1% (19/24) of patients who underwent video-assisted thoracoscopic surgery lobectomy for lung cancer. Wang et al. (20) defined preoperative sleep disturbance as a PSQI score ≥7 and reported that its incidence was 47.8% in cancer patients scheduled for elective surgery (142/297). Yang et al. (21) used the insomnia severity index to assess sleep quality in patients who underwent elective spine surgery; the authors reported that the prevalence of preoperative clinical insomnia (insomnia severity index ≥15) was 49.7% (109/219). The current study enrolled 563 patients who underwent surgery, including 111 (19.7%) patients with surgical site on head and neck, 102 (18.1%) on the chest, 124 (22.0%) on the abdomen, and 226 (40.1%) on the pelvic cavity. Our study gathered sleep quality data and revealed that the prevalence of preoperative acute insomnia was 78.5% (442/563). Compared with previous studies, our study had a larger sample size and enrolled patients who underwent different types of surgery (4, 6, 19–21). These data indicate that preoperative acute insomnia is a pervasive issue and should receive increased attention.

Through association analyses, it was revealed that age, AHI score, and AHI stage were positively while education level was negatively associated with preoperative acute insomnia. Age is widely recognized to be associated with insomnia (22), and our findings are in good agreement with this opinion. The AHI score and AHI stage reflect the risk of obstructive sleep apnea, and the latter might severely affect sleep quality, leading to preoperative acute insomnia (23, 24). With respect to education level, previous studies have revealed that a lower education level is associated with insomnia, and our findings are in accordance with these previous studies (25, 26).

HRV characteristics reflect the activation of the sympathetic and parasympathetic systems, as well as the balance between them (9). The current study revealed that LF and HF were both positively associated with preoperative acute insomnia. LF reflects the activation of both the sympathetic and parasympathetic systems, whereas HF reflects the activation of the parasympathetic system (27). Therefore, the findings of our study suggested that activation of the parasympathetic system was associated with preoperative acute insomnia. Hyperarousal is proposed to be involved in the development of insomnia (28). According to a previous study, activation of the parasympathetic system was associated with hyperarousal (29). Thus, the findings of our study supported that the activation of the parasympathetic system induced hyperarousal, which further contributed to preoperative acute insomnia. However, our findings were contradictory to previous studies, which reported that lower HF was associated with insomnia (10, 11). A possible explanation was that our study assessed preoperative acute insomnia, and patients in previous studies were confronted with chronic insomnia (10, 11). Before surgery, patients could experience acute insomnia due to anxiety and unfamiliarity with the environment in the hospital, and the activation of the parasympathetic system might be a result of the antagonism of acute insomnia. In patients with chronic insomnia, insufficient activation of the parasympathetic system leads to the release of adrenaline, causing insomnia (30). However, further exploration is warranted for verification and investigation of potential physiological reasons.

The present study used multivariate logistic regression analyses and screened out education level, AHI score, SDNN, rMSSD, pNN50, ULF, LF/HF ratio, and the HF ratio to construct a predictive model for preoperative acute insomnia. The performance of the predictive model was assessed by ROC analysis, which showed an AUC of 0.750, indicating the good predictive value of this model. We also presented a predictive model using a nomogram. Nomograms are powerful tools for risk prediction and are characterized by easy performance and intuitive information display (31). In this study, the nomogram for predicting the risk of preoperative acute insomnia was composed of education level, AHI score, and HRV characteristics. The education level of patients can be easily acquired, and the AHI score and HRV characteristics can be generated by the CPC, which is also feasible in clinical practice. According to the nomogram, the sum of each parameter could be projected to indicate the risk of preoperative acute insomnia, which is easy to use. According to previous studies, preoperative acute insomnia exacerbates surgery-induced neuroinflammation and neuronal damage and is associated with postoperative pain and postoperative delirium, which strongly affects the recovery and quality of life of patients (3–5). By using this predictive tool, clinicians might predict the risk of preoperative acute insomnia quickly. For patients at high risk of preoperative insomnia, targeted interventions could be applied, such as more intensive postoperative pain management and postanaesthesia monitoring, as well as anti-inflammatory treatment, thus improving the management of these patients.

The highlights of this study are that we investigated the associations of HRV characteristics with preoperative acute insomnia and then established a predictive model for preoperative acute insomnia on the basis of HRV characteristics and demographics. We also constructed a nomogram based on the predictive model, by which clinicians could predict the risk of preoperative acute insomnia quickly. To date, no published studies have reported the associations between HRV characteristics and preoperative acute insomnia. This study provides a novel perspective that preoperative acute insomnia is associated with an imbalance in the sympathetic and parasympathetic systems and provides a tool for predicting preoperative acute insomnia, which could help improve the management of this syndrome.

Several limitations should be clarified. First, this was a single-center study, selection bias was inevitable, and the findings of this study might not be applicable to patients from other regions. Second, this study did not include a validation cohort to further explore the performance of the predictive model for the risk of preoperative acute insomnia. Third, this study did not exclude patients with preexistence of obstructive sleep apnea, which might potentially affect the findings of this study, leading to overestimation of the incidence of preoperative acute insomnia. Fourth, the predictive model for preoperative acute insomnia lacked external or internal validation. Further studies should consider validating this model. Fifth, this study did not investigate the influence of minor or major surgery, differences in underlying disorders, or potential prognoses on preoperative acute insomnia. Further studies could explore this issue further. Sixth, specific postsurgery outcomes were not collected, and whether HRV characteristics or preoperative acute insomnia could predict postsurgery outcomes should be investigated in the future. Seventh, some patients might have insomnia due to other medical or psychological disorders. However, the current study could not discriminate and exclude these patients. As a result, the findings of this study could be influenced.

Conclusively, preoperative acute insomnia is a prevalent issue and is associated with an imbalance of the sympathetic/parasympathetic system. The current study also constructed a predictive model for preoperative acute insomnia, which could improve the management of this symptom.

## Data Availability

The original contributions presented in the study are included in the article/supplementary material, further inquiries can be directed to the corresponding authors.
